# Comparison of radiation doses using weight-based protocol and dose modulation techniques for patients undergoing biphasic abdominal computed tomography examinations

**DOI:** 10.4103/0971-6203.56085

**Published:** 2009

**Authors:** Roshan S. Livingstone, Paul M. Dinakaran, Rekha S. Cherian, Anu Eapen

**Affiliations:** Department of Radiology, Christian Medical College, Vellore - 632 004, TN, India

**Keywords:** Abdominal computed tomography, dose modulation, effective dose, image quality

## Abstract

Computed tomography (CT) of the abdomen contributes a substantial amount of man-made radiation dose to patients and use of this modality is on the increase. This study intends to compare radiation dose and image quality using dose modulation techniques and weight- based protocol exposure parameters for biphasic abdominal CT. Using a six-slice CT scanner, a prospective study of 426 patients who underwent abdominal CT examinations was performed. Constant tube potentials of 90 kV and 120 kV were used for all arterial and portal venous phase respectively. The tube current-time product for weight-based protocol was optimized according to patient's body weight; this was automatically selected in dose modulations. The effective dose using weight-based protocol, angular and z-axis dose modulation was 11.3 mSv, 9.5 mSv and 8.2 mSv respectively for the patient's body weight ranging from 40 to 60 kg. For patients of body weights ranging 60 to 80 kg, the effective doses were 13.2 mSv, 11.2 mSv and 10.6 mSv respectively. The use of dose modulation technique resulted in a reduction of 16 to 28% in radiation dose with acceptable diagnostic accuracy in comparison to the use of weight-based protocol settings.

## Introduction

The role of computed tomography (CT) has been of tremendous value since its inception in diagnostic radiology. Though other imaging modalities such as MRI and ultrasonography are widely used in the present scenario, despite the large radiation dose imparted to patients, CT imaging continues to be on the increase due to its varied advantages. In the last decade there has been a corresponding increase in the number of CT examinations performed around the world.[1] The advent of multislice CT (MSCT) makes possible rapid volume acquisition and has opened new diagnostic fields such as CT angiography and CT colonography.[2] Due to its widespread use, CT contributes a large fraction of man-made radiation dose to the human population and radiation dose to patients from this modality should be optimized.[3,4] Generally, the radiation doses to patients are about 30 to 50% greater with the use of MSCT as a result primarily of scan overlap, positioning of the x-ray tube closer to the patient, overbeaming, increased significance of overscanning and possibly increased scattered radiation with wider x-ray beams.[5,6] Absence of optimization in MSCT may well be significantly higher than their single slice counterparts. According to literature, the risk for radiation-induced cancer from CT examinations to patients is not negligible.[7–11] Effective dose from CT of abdomen is 10 mSv and has an associated risk of 1 in 2000.[12]

For scanning in large patients, radiation dose must be increased to obtain diagnostic quality images. There has been a tendency to increase the tube current-time product to avoid excessive noise on images, particularly for large patients and at thin-section CT, which is more readily available with the newer generations of scanners. A reduction in the radiation dose delivered from CT has become an important issue and various dose reduction and optimization techniques have been formulated.[13,14] Modulation of the x-ray tube current during scanning is one effective method of managing the dose.[13] Automatic tube current modulation in CT is analogous to the automatic exposure control (AEC) or photo timing technique used for automatically terminating radiographic exposure in conventional radiography once the predetermined radiographic density has been obtained.[15] The techniques used are angular (x and y-axis) and z-axis tube current modulations. The x and y-axis modulation involves variation in tube current as the x-ray tube rotates about the patient, while the z-axis modulation involves variation in tube current along the z-axis of the patient.[13] The current study intends to compare radiation dose and image quality achieved with weight-based protocol, along with the dose modulation software available in the machine i.e. dynamic dose modulation (D-DOM) and z-axis dose modulation (Z-DOM) dose modulation techniques using a six-slice CT scanner. The D-DOM and Z-DOM are based on the angular and z-axis tube current modulation respectively.

## Materials and Methods

A prospective study involving 426 patients who underwent biphasic abdominal CT was performed using a six-slice CT scanner (Brilliance, Philips medical systems, Netherlands). The tube potential, tube current-time product, volumetric CT dose index (CTDI_vol_) and dose length product (DLP) values were displayed on the console of the scanner. The tube potential available in the machine was 90kV, 120 kV and 140 kV. Various other parameters such as the total time duration of the scan, field of view and pitch selection were displayed on the console. The scanner facilitated preprogrammed protocols designed for quick and easy workflow. These protocols involved a complete examination of the region of interest along with a topogram, spiral or sequential ranges and reconstruction modes. The preprogrammed scan protocols used were based on recommended exposure factors specified by the manufacturers as a starting point for clinical work. During the course of the study, exposure parameters were selected according to the patient's body weights and were lower than the preset protocols. This study was a part of the project funded by the atomic energy regulatory board (AERB) of India and was carried out after an ethical committee clearance from the institution.

Constant tube potentials of 90 kV and 120 kV were used for all arterial and portal venous phases respectively. A tube current-time product of 250 mAs and 180 mAs for arterial and portal venous phase was used for patients with body weight between 40 to 60 kg (mean 50.8 kg). A 10% increase of tube current-time product for both arterial and portal venous phase was used for patients with body weight between 61 to 80 kg (mean 68.3 kg). The increase in the tube current-time product was to reduce noise in the images when large patients were scanned. The tube potentials for D-DOM and Z-DOM were also kept constant similar to weight based protocol; however, the tube current-time products were automatically selected by the scanner.

### Radiation Dose Measurement

The CTDI_vol_ and DLP values available from the CT console were recorded and entered into a database. The database also included the exposure parameters, age, sex, weight, scan length for arterial and portal venous phase and number of sections acquired. Periodic calibrations using 32 cm polymethyl methacrylate (PMMA) CTDI body phantom with a high sensitivity 100 mm long pencil ion chamber (CTDI_100_, Victoreen, Ohio, USA) were performed to check the consistency of the CTDI_w_ values. Phantom measurements were made at the centre (CTDI_100,c_) and periphery (CTDI_100,p_) and was used to calculate the CTDI_w_ values.[16] The weighted CT dose index (CTDI_w_) values were obtained using the formula

CTDIw=1/3CTDI100,c+2/3CTDI100,p

The DLP was obtained using scan length and beam pitch. The effective doses were estimated by multiplying the DLP values by normalized coefficients found in the European guidelines on quality criteria of CT which is 0.015 mSv mGy^−1^ cm^−1^.[17]

### Image Quality Analysis

Objective evaluation of image quality was based on evaluating CT image noise of the uniformly attenuating region of the liver. For this purpose, the signal to noise ratio (SNR) was measured in a standard one-cm^2^ circular region of interest (ROI) from the CT console. The ROI was selected such a way that the region did not have any undue influence from contrast in the blood vessels. One radiologist rated the randomized CT scans for overall image quality and anatomic details of liver, spleen, adrenal glands, kidneys, pancreas, and abdominal wall using a 5 point scale (1 = unacceptable, 2 = substandard, 3 = acceptable, 4 = above average, and 5 = superior).[18] The anonymity of the exposure parameters was maintained and was not revealed on the workstation which the radiologist used to review images. The overall average image quality scores for different body weights for weight based protocol, D-DOM and Z-DOM were marked by the radiologist.

### CT Clinical Examination

The biphasic abdominal CT examination involved administration of oral and intravenous contrast agents to patients. A 30 ml sodium meglumine diatrizoate solution (gastroscan) dissolved in one liter of water was administered as an oral contrast one hour prior to the scan. Acquisition of images in CT was preceded by injecting 100 ml iopamidol (Iopamiro 370, Bracco) of iodinated contrast material intravenously using remote pressure injector and a bolus tracking was done using CT sections in the region of the aorta. Once the contrast media reached the aorta, a 5 mm section thickness arterial phase starting from the domes of the diaphragm covering the entire liver followed by a 5 mm portal venous phase with a delay of 40 seconds starting from lobes to the pelvis was acquired. A nominal pitch of 1.2 with gantry rotation time of 0.75 second, beam collimation of 6 × 3mm and matrix size of 512 × 512 pixels was invariably selected for the biphasic study. The reconstructed slice widths were also 5 mm. The acquired images were archived to the picture archival and communication systems (PACS) in digital images and communication in medicine (DICOM) format. These images were viewed using high resolution monitors which were linked to the work stations in PACS.

## Results

Of all the 426 patients who had undergone biphasic abdominal examination, 233 were males and 193 were females. The number of sections acquired varied from 24 to 66 for the arterial phase and 64 to 121 for the portal venous phase. The selection of the number of sections depended upon the anatomy of patients involved in the study. Table 1 shows scan lengths of patients according to the respective body weights for arterial and portal venous phases. The maximum length scanned in the arterial phase and portal venous phase was 330 mm and 570 mm respectively.

**Table 1:** Scan length and number of sections acquired in biphasic abdominal CT

*Patients body weight in Kg*	*Mean Scan length in mm (range)*		*Mean Number of slices (range)*	
		
	*Arterial*	*Portal venous phase*	*Arterial*	*Portal venous phase*
40 - 60	188 (105 - 265)	409 (320 - 560)	38 (24 - 53)	82 (64 -121)
61 - 80	197 (145 - 330)	434 (365 - 570)	39 (28 - 66)	87 (73 - 114)
81 and above	205 (160 - 250)	458 (410 - 545)	41 (32 - 50)	92 (82 - 109)

Table 2 shows the exposure parameters used during weight-based protocol, D-DOM and Z-DOM for various patient body weights. A reduction of current-time product of approximately three to five percent using D-DOM and 37 to 55% using Z-DOM was achieved for arterial and portal venous phases compared to the weight based protocol settings. A reduction of approximately 30 to 50% of tube current-time product was noted within D-DOM and Z-DOM respectively for arterial and portal venous phases.

**Table 2:** Exposure parameters in weight-based protocol, D-DOM and Z-DOM

*Weight in kg*		*Mean exposure parameters*												
			
	*Weight based protocol setting*				*D-DOM*				*Z-DOM*			
			
	*Arterial phase*		*Portal venous phase*		*Arterial phase*		*Portal venous phase*		*Arterial phase*		*Portal venous phase*	
						
	*kV*	*mAs*	*kV*	*mAs*	*kV*	*mAs (range)*	*kV*	*mAs (range)*	*kV*	*mAs (range)*	*kV*	*mAs (range)*
40 - 60	90	250	120	180	90	243 (235 - 248)	120	175 (169 - 178)	90	158 (88 - 292)	120	82 (41 - 189)
61 - 80	90	275	120	200	90	267 (256 - 273)	120	194 (188 -197)	90	191 (133 - 313)	120	107 (70 - 212)
81 and above	90	295	120	225	90	282 (272 - 292)	120	215 (208 - 220)	90	195 (107 - 312)	120	118 (67 - 264)

Tables 3–5 show the CTDI_vol_, DLP and mean effective doses for weight based protocol, D-DOM and Z-DOM. The mean effective doses reported in these tables contributions from both arterial and venous phases. The CTDI_vol_ values for weight based protocol settings were constant while it varied for D-DOM and Z-DOM. The planned CTDI_vol_ values as seen on the console were three to five percent higher than the actual values recruited by the machine for arterial and portal venous phase respectively in using D-DOM technique. The planned CTDI_vol_ values were 34 and 51% higher than Z-DOM technique used by the machine for arterial and portal venous phase respectively. Reduction of effective dose for patients weighing 40 to 60 kg would be 28% with the use of Z-DOM technique in comparison with weight based protocol settings. Similarly a reduction of 14% in effective dose is noted between D-DOM and Z-DOM for patients weighing 40 to 60 kgs. A reduction of 24% in effective dose was noted between weight based protocol and Z-DOM settings for patient's of body weight higher than 80 kgs.

**Table 3:** CTDI_vol_, DLP and effective dose values for abdominal CT examination using weight-based protocol

*Weight in kg*	*No. of cases*	*Weight based protocol*						*Mean Effective dose mSv ± SD (range)*

		*Arterial phase*			*Portal venous phase*			
	
		*Mean CTDI_vol_ (mGy)*	*Mean DLP (mGy cm) (range)*	*Mean Effective dose (mSv) ± SD (range)*	*Mean CTDI_vol_ (mGy)*	*Mean DLP (mGy cm) (range)*	*Mean Effective dose (mSv) ± SD (range)*	
40 - 60	71	7.9	174 (138 - 218)	2.6 ± 0.25 (2.1 - 3.3)	13.2	581 (495 - 655)	9 ± 0.55 (7.4 - 9.8)	11.3 ± 0.69 (9.5 - 13.1)
61 - 80	53	8.7	202 (153 - 312)	3 ± 0.37 (2.3 - 4.7)	14.6	677 (628 - 796)	10 ± 0.55 (9.4 - 11.9)	13.2 ± 0.72 (11.7 - 16.6)
81 and above	5	9.3	217 (196 - 233)	3.3 ± 0.2 (2.9 - 3.5)	16.5	834 (743 - 916)	13 ± 1.21 (11 - 13.7)	16 ± 1.24 (13.9 - 17.2)

* SD - Standard deviation

**Table 4:** CTDI_vol_, DLP and effective dose values for abdominal CT examination using D-DOM

*Weight in kg*	*No. of cases*	*D-DOM*						*Mean effective dose mSv ± SD (range)*

		*Arterial phase*			*Portal venous phase*			
	
		*Mean CTDI_vol_ (mGy) (range)*	*Mean DLP (mGy cm) (range)*	*Mean effective dose (mSv) ± SD (range)*	*Mean CTDI_vol_ (mGy) (range)*	*Mean DLP (mGy cm) (range)*	*Mean effective dose (mSv) ± SD (range)*	
40 - 60	93	7.7 (6.7 -7. 8)	151 (119 - 198)	2.3 ± 0.22 (1.78 - 2.97)	12.8 (12.3 - 13.1)	484 (406 - 605)	7.3 ± 0.53 (6.1 - 9.1)	9.5 ± 0.68 (7.9 - 12.1)
61 - 80	68	8.4 (7.6 - 8.6)	172 (134 - 210)	2.6 ± 0.26 (2 - 3.2)	14.1 (13.6 - 14.4)	575 (512 - 741)	8.62 ± 0.67 (7.68 - 11.11)	11.2 ± 0.8 (9.68 - 14.3)
81 and above	12	8.9 (8.5 - 9.2)	191 (157 - 225)	2.9 ± 0.24 (2.4 - 3.4)	15.7 (15.2 - 16.1)	684 (601 - 816)	10.3 ± 0.92 (9.01 - 12.25)	13.1 ± 1.05 (11.4 - 15.7)

* SD - Standard deviation

**Table 5:** CTDI_vol_, DLP and effective dose values for abdominal CT examinations using Z-DOM

*Weight in kg*	*No. of cases*	*Z-DOM*						*Mean Effective dose mSv ± SD (range)*

		*Arterial phase*			*Portal Venous phase*			
	
		*Mean CTDI_vol_ (mGy) (range)*	*Mean DLP (mGy cm) (range)*	*Mean Effective dose (mSv) ± SD(range)*	*Mean CTDI_vol_ (mGy)(range)*	*Mean DLP (mGy cm)(range)*	*Mean Effective dose (mSv) ± SD (range)*	
40 - 60	71	5 (2.8 - 10.1)	137 (82 - 212)	2.1 ± 0.5 (1.22 - 3.2)	6 (3 - 13.8)	411 (208 - 680)	6.2 ± 1.32 (3.12 - 10.20)	8.2 ± 1.7 (4.3 - 13.4)
61 - 80	41	6.2 (4.2 - 9.9)	175.2 (113 - 284)	2.63 ± 0.5 (1.7 - 4.26)	7.7 (5.1 - 15.1)	531 (338 - 742)	8 ± 1.3 (5.06 - 11.13)	10.6 ± 1.6 (6.8 - 15.4)
81 and above	12	6 (3.3 - 9.8)	183.2 (118 - 278)	2.8 ± 0.7 (1.76 - 4.17)	8.6 (5 - 19.3)	618 (414 - 871)	9.3 ± 2.1 (6.21 - 13.06)	12 ± 2.7 (7.8 - 17.23)

* SD - Standard deviation

From Table 6 it is evident that the SNR measured with a one-cm^2^ region of interest for D-DOM and Z-DOM were not significantly different for arterial phase (p = 0.097). The SNR values for D-DOM and weight based protocol were not statistically significant for portal venous phase (p = 0.14). The arterial phase SNR value comparisons between weight based protocol and D-DOM (p = 0.001) and weight based protocol and Z-DOM were significantly different (p < 0.001). The portal venous phase comparisons between weight based protocol and Z-DOM and D-DOM and Z-DOM were significantly different with p < 0.001 for each technique respectively. The statistical test of significance used was a one-way analysis of variance (ANOVA). An acrylic body phantom was used to analyze the image quality and the CT number. The mean CT number for the acrylic phantom was 112 HU and was within the range recommended by the ACR CT phantom testing criteria for acrylic (110 to 130).[19]

**Table 6:** Image quality assessment for arterial and portal venous phases for weight based protocol, D-DOM and Z-DOM

*Patient body weight*	*Signal to Noise values*						
		
	*Weight based protocol*		*D-DOM*		*Z-DOM*	
			
	*Arterial phase*	*Venous phase*	*Arterial phase*	*Venous phase*	*Arterial phase*	*Venous phase*
40 - 60 kg	7.69 (4.5 -13.3)	6.22 (4.5-10.7)	8.3 (4.9-11.6)	6.46 (4.1-9)	8.5 (6.5-11.5)	7.9 (6.4-9.8)
61 - 80 kg	9.13 (5.8-15.7)	7.36 (5.4-11)	9.93 (6.6-13.8)	7.57 (5.4-11.1)	10.61 (8.7-15.2)	9.27 (7.6-10.8)
81 and above	10.48 (8.4-12.5)	7.38 (5.5-8.5)	13.25 (10.5-15.6)	8.68 (7.9-9.5)	15.05 (12.7-20.1)	11.79 (9.3-14.8)

One radiologist with eight years experience in abdominal CT, independently performed a blind qualitative analysis of CT images obtained with each individual. The median value for assessing image quality for liver, kidneys, pancreas, spleen, gall bladder and muscle for both arterial and portal venous phase was found to be 5 (superior) for weight-based protocol and D-DOM. The median value for assessing image quality using Z-DOM was found to be 4 (above average) for kidney and pancreas in the portal venous phase and for gall bladder in the arterial phase. However, median value of 5 was assigned for the rest of the organs.

## DISCUSSION

There is a risk of imparting high radiation doses to patients during examinations performed using CT with multiple exposures inherent in the examination. Modern CT scanners are versatile in their operation and with a wide range of facilities available on these scanners; there is an increasing need to assess the dose delivered during routine CT examinations[20]. The results of this study show that the mean effective dose imparted to patients using weight-based protocol is a factor of 1.2 to 1.4 times higher than that with the use of Z-DOM. Dose relevant parameters such as exposure parameters and scan length differed for the biphasic study. Therefore, the DLP was calculated separately for each scan series. The total radiation exposure for the complete examination was obtained by adding the contributions from each phase.

During arterial phase, a tube potential of 90 kV was selected since the area of scanning was restricted to the upper abdomen covering the entire liver which was more of soft tissue; tube potential of 120 kV was used for scanning in the portal venous phase from the domes of the diaphragm to below the pubic symphysis, since this region included bony interfaces. With the use of this technique, a reduction of doses up to 44% can be achieved [table 3]. As reported by Nakayama *et al*. the reduction of tube voltage from 120 kV to 90 kV can reduce the amount of contrast material to at least 20% without degradation in image quality. Study findings confirmed that in scans obtained with low tube voltage, the radiation dose reduced by as much as 57% and these scans yielded higher contrast material enhancement.[2] It is noteworthy in this context, that the use of D-DOM and Z-DOM in combination with these tube potentials will deliver doses much lower than the weight-based protocol. The effective doses for weight-based protocol, D-DOM and Z-DOM, were significantly different (p < 0.001).

Though there are possibilities of reduction in effective doses using dose modulation techniques, the use of Z-DOM imparted a highest dose of 17.3 mSv for a patient weighing 113 kg. An effective dose of 17 mSv with the use of weight-based protocol was recorded for a patient weighing 92 kg. The increase of dose may be operator dependent if the scan length is increased for some patients to see the pathology and abnormality. The effective doses reported in the current study ranged from 4.3 mSv to 17.3 mSv with and without the use of dose modulation techniques were lower than the effective dose range of 6 to 24 mSv for abdominal CT reported by Tsapaki *et al*.[21]. Goddard and Al-Farsi reported mean effective dose for CT of abdomen of 9.5 mSv (1.4 to 31.2 mSv) for abdominal CT.[22] Van Der Molen *et al*, reported an effective dose of 3.3 mSv for arterial phase and 6.9 mSv for portal venous phase for CT abdomen performed using 16 slice CT scanner.[23] The effective dose reported by Brix *et al*. and Shrimpon *et al*. were 15.2 mSv (arterial phase of 5.5 mSv and portal phase of 9.7 mSv) and 12.5 mSv (3.9 mSv and 8.6 mSv) respectively.[24,25] These values were higher than those used in the present study for patients' weight ranging from 40 to 60 kg. However, effective doses reported by these studies were similar to values from the present study for patients' weight ranging from 60 and above. In the current study, radiation dose from topogram and when involved with bolus tracking was found to be 0.06 mSv which would be equivalent to dose contributed from three chest radiographs (0.02 mSv).[26]

The preprogrammed exposure parameters set in the machine may tend to impart high radiation dose to patients if adequate optimization is not performed. Hence it is prudent to adopt Z-DOM techniques so that the doses are as low as reasonably achievable. Karla *et al*, report that “In z-axis modulation, tube current is adjusted to maintain a user-selected image quality level in the image data. Noise is regulated on the final image to a level desired by the user. Z-axis modulation is an attempt to render all images with similar noise, independent of patient size and anatomy”.[28] Some of the software techniques which can further improve CT image quality using low dose protocols are reported in literature.[29,30] These techniques can be implemented along with dose modulation software to provide adequate image quality. Images acquired using low dose protocols should be reviewed by a team of expert radiologists and put into regular practice. Radiologists are responsible for medical radiation doses to their patients, and it is imperative that they understand the relationship between radiation dose and image quality.[26]

In conclusion, the results from this study show that dose reduction of 16 to 28% was possible with the use of tube current modulation techniques without sacrificing diagnostic image quality in comparison to selection of weight-based protocol. Dose reduction is possible with the modern CT scanners if proper work practices are followed by personnel operating the machine. The dose modulation technique is an effective method to manage dose to patients undergoing CT examinations when compared to the weight-based protocol.

## References

[1] Mulkens TH, Bellinck P, Baeyaert M, Ghysen D, Van Dijck X, Mussen E (2005). Use of an automatic exposure control mechanism for dose optimisation in multi-detector row CT examinations: Clinical evaluation. Radiology.

[2] Nakayama Y, Awai K, Funama Y, Hatemura M, Imuta M, Nakaura T (2005). Abdominal CT with low tube voltage: Preliminary observations about radiation dose, contrast enhancement, image quality, and noise. Radiology.

[3] Brenner DJ, Hall EJ (2007). Computed tomography: An increasing source of radiation exposure. N Engl J Med.

[4] Rehani MM, Berry M (2000). Radiation doses in computed tomography: The increasing doses of radiation need to be controlled. BMJ.

[5] McCollough CH, Zink FE (1999). Performance evaluation of a multi-slice CT system. Med Phys.

[6] Hidajat N, Maurer J, Schroder RJ, Wolf M, Vogl T, Felix R (1999). Radiation exposure in spiral computed tomography: Dose distribution and dose reduction. Invest Radiol.

[7] Brenner D, Elliston C, Hall E, Berdon W (2001). Estimated risks of radiation-induced fatal cancer from pediatric CT. AJR Am J Roentgenol.

[8] Brenner DJ, Elliston CD (2004). Estimated radiation risks potentially associated with full body CT screening. Radiology.

[9] Brenner DJ (2004). Radiation risks potentially associated with low-dose CT screening of adult smokers for lung cancer. Radiology.

[10] Yoshinaga S, Mabuchi K, Sigurdson AJ, Doody MM, Ron E (2004). Cancer risks among radiologists and radiologic technologists: Review of epidemiologic studies. Radiology.

[11] Wagner LK (2004). Overconfidence, overexposure, and overprotection. Radiology.

[12] Hart D, Hillier MC, Wall BF (2002). Doses to patients from medical x-ray examinations in the UK - 2000 review. Chilton.

[13] McCollough CH, Bruesewitz MR, James M, Kofler T (2006). Dose reduction and dose management tools: Overview of available options 1. RadioGraphics.

[14] Tack D, Maertelaer VD, Gevenois PA (2003). Dose reduction in multidetector CT using attenuation-based online tube current modulation. AJR Am J Roentgenol.

[15] Rizzo S, Kalra M, Schmidt B, Dalal T, Suess C, Flohr T (2006). Comparison of angular and combined automatic tube current modulation techniques with constant tube current CT of the abdomen and pelvis. AJR Am J Roentgenol.

[16] Huda W, Nickoloff EL, Boone JM (2008). Overview of patient dosimetry in diagnostic radiology in the USA for the past 50 years. Med Phys.

[17] European Commission (1999). European guidelines on quality criteria for computed tomography.

[18] Kalra MK, Prasad S, Saini S, Blake MA, Varghese J, Halpern EF (2002). Clinical comparison of standard- dose and 50% reduced-dose abdominal CT: Effect on image quality. AJR Am J Roentgenol.

[19] ACR (1891). Computed Tomography Accreditation program: Phantom testing criteria. Preston White Drive, Reston VA20191.

[20] Wade JP, Weyman JC, Goldstone KE (1997). CT standard protocols are of limited value in assessing actual patient dose. Br J Radiol.

[21] Tsapaki V, Aldrich JE, Sharma R, Staniszewska MA, Krisanachinda A, Rehani M (2006). Dose reduction in CT while maintaining diagnostic confidence: Diagnostic reference levels at routine head, chest, and abdominal CT-IAEA-coordinated research project. Radiology.

[22] Goddard CC, Al-Farsi A (1999). Radiation doses from CT in the Sultanate of Oman. BJR.

[23] Van der Molen AJ, Veldkamp WJ, Geleijns J (2007). 16-slice CT: Achievable effective doses of common protocols in comparison with recent CT dose surveys. Br J Radiol.

[24] Brix G, Nagel HD, Stamm G, Veit R, Lechel U, Griebel J (2003). Radiation exposure in multi-slice versus single-slice spiral CT: Results of a nationwide survey. Eur Radiol.

[25] Shrimpton PC, Hillier MC, Lewis MA, Dunn M Doses from computed tomography examinations in the UK - 2003 review. Report NRPB-W67.

[26] Wall BF, Hart D (1997). Revised radiation doses for typical x-ray examinations. Br J Radiol.

[27] Kalra MK, Maher MM, Toth TL, Hamberg LM, Blake MA, Shepard JA (2004). Strategies for CT radiation dose optimisation. Radiology.

[28] Huda W, Ravenel JG, Scalzetti EM (2002). How do radiographic techniques affect image quality and patient doses in CT?. Semin Ultrasound CT MR.

[29] Borsdorf A, Raupach R, Flohr T, Hornegger J (2008). Wavelet based noise reduction in CT-images using correlation analysis. IEEE Trans Med Imaging.

[30] Wang J, Li T, Lu H, Liang Z (2006). Noise reduction for low-dose single slice helical CT sinograms. IEEE Trans Nucl Sci.

